# Game Related Statistics Discriminate National and Foreign Players According to Playing Position and Team Ability in the Women’s Basketball EuroLeague

**DOI:** 10.3390/ijerph17155507

**Published:** 2020-07-30

**Authors:** Lorenzo Gasperi, Daniele Conte, Anthony Leicht, Miguel-Ángel Gómez-Ruano

**Affiliations:** 1 Facultad de Ciencias de la Actividad Física y del Deporte, Universidad Politécnica de Madrid, 28031 Madrid, Spain; miguelangel.gomez.ruano@upm.es; 2 Institute of Sport Science and Innovations, Lithuanian Sports University, 44221 Kaunas, Lithuania; daniele.conte@lsu.lt; 3 Sport and Exercise Science, James Cook University, Townsville 4814, Australia; anthony.leicht@jcu.edu.au

**Keywords:** game-related statistics, team sports, discriminant scores, player position, team ability, foreign players, women basketball

## Abstract

The aim of the present study was to examine the differences in game-related statistics between national and foreign female basketball players in the Women’s EuroLeague, according to playing positions and team ability. The official box-scores of 112 games from the 2016–2017 season of the Women’s EuroLeague (FIBA) were examined. Players were categorised based upon country of nationality versus competition (i.e., foreign or national), playing positions (i.e., Guards, Forwards, Centers), and team ability (i.e., four groups using a cluster of k-means analysis according to the winning percentage of each team during the competition). A structural coefficient (SC) above |0.30| was used to identify the variables that best differentiated the national and foreign players. Results showed that foreign players had a better performance according to team ability and playing position for most of the performance indicators, with higher values for minutes played, percentage of successful 2-point field-goals, percentage of successful free-throws, and percentage of assists. Moreover, foreign players performed better in variables associated with offensive situations, while national players were prevailing with indicators associated with defensive actions. These results have highlighted the unique contributions of foreign and national players, based upon playing position and team ability, to team success in the Euroleague. This information will assist the recruitment process of national and foreign athletes for coaches to develop successful elite female basketball teams.

## 1. Introduction

Basketball is a sport that encompasses interactions between physiological fitness, psychological preparedness, physical development, biomechanical proficiency, and tactical awareness, amongst several others [1]. Previous research investigating performance indicators in female basketball have provided a clear understanding of game performance requirements [2,3,4]. Defensive and offensive rebounds, successful 3-point and 2-point field-goals, assists, and inside game play tactics were reported to be the key performances indicators (KPIs) in female basketball as they increased the possibility of winning at national and international tournaments [5,6]. Further, situational variables such as team ability and player’s position were identified as important factors for game performances [4,7,8,9]. For example, team ability, classified by the percentage of winning games during a basketball season [8,10], was able to differentiate professional male teams in terms of game statistics (e.g., 2-point field-goals, passes, and defensive rebounds), with lower performances for teams with low team ability [8]. For playing position, Forwards were demonstrated to exhibit greater shooting efficacy inside the paint, which was more important to match outcome than that of other playing positions such as Guards and Centers [11]. However, these variables were mainly investigated in male basketball performances [4], while little information was available for female basketball players. Additionally, no previous study has assessed these variables during a female basketball competition, with further studies addressing the effect of team ability and playing position on basketball game-related statistics needed.

Understanding the performance profile of female basketball players in accordance with contextual factors is vital for teams to optimize recruitment of players (i.e., searching, scouting, and signing players). Female basketball players usually change teams, countries, and/or continents during their career based upon finding the team best suited to their playing characteristics and/or financial rewards [12]. Therefore, players will likely compete in their home nation as well as in foreign countries [13]. For example, the number of foreign players engaged with the National Basketball Association (NBA), which is the premier basketball league in the world, increased from 35 in 1999 to 110 in 2018 [14]. Similarly, the number of foreign players in the Women’s NBA (WNBA) increased from 30 in the 2017/2018 season to 38 in the 2018/2019 season (information obtained from https://en.wikipedia.org/wiki/List_of_foreign_WNBA_players). This growth in foreign players may be driven by profit (e.g., expanding the team brand, followed by signing foreign top players) and/or performance by incorporating the most skilled players for greater team performance [14]. Therefore, the inclusion of both national and foreign players may affect team performance, with this impact unknown within female basketball competitions. Although, some evidence does exist for male basketball competitions, with the performance efficiency of foreign players across several seasons (from 1997 to 2010) within the Turkish Basketball League greater compared to native players [15]. Therefore, examination of female players’ nationality in conjunction with contextual variables such as playing position and team ability may assist coaches to gain a better understanding of players’ individual contribution to overall team performances [16]. Further, this information could assist coaches, managers, and performance analysts with effective decisions during recruitment processes. Therefore, the aim of the present study was to examine the differences in game-related statistics or performance between national and foreign basketball players in an elite female competition (EuroLeague), according to playing position and team ability.

## 2. Materials and Methods

### 2.1. Data

This study was a retrospective analysis of publically available data obtained from the 2016–2017 season, official box-scores of the FIBA Women’s EuroLeague. Data obtained using this methodology across different contexts has exhibited good reliability [17,18]. All games from the initial group phase (n = 112 games) were selected with the following game-related statistics gathered: 2- and 3-point field-goal shots (both successful and unsuccessful), free-throw shots (both successful and unsuccessful), defensive and offensive rebounds, blocks, assists, fouls, steals, turnovers, and minutes played. These metrics have been examined routinely in prior studies of basketball player and team performances [8,11]. 

Players were categorized as either national players (i.e., athletes playing for a club competing in the national competition of the nation of their own nationality) or foreign players (i.e., athletes playing for a club competing in a national competition that is different from the nation of their own nationality). For the 200 players within the 16 women’s Basketball Euroleague teams, 119 were national players (59.5% of the total) and 81 were foreign players (40.5% of the total). For the 112 games examined in the current study that resulted in 2225 player observations, 1159 observations were from national players’ (52.1% of the total) and 1066 observations were from foreign players (47.9% of the total). Regarding Guards, 943 player observations included 528 from national players (56.0% of the total) and 415 were from foreign players (44.0% of the total). Regarding Forwards, 539 observations were from 287 national players (53.3% of the total) and 252 were from foreign players (46.7% of the total). Regarding Centers, 743 observations were from 344 national players (46.3% of the total) and 399 were from foreign players (53.7% of the total) [8]. Moreover, team ability was determined by dividing the sample into four groups using a k-means cluster analysis according to the winning percentage of each team during the competition [winning % = (games won/total games) * 100]. The four groups based upon team ability were poor (11.2 ± 3.4%), low (42.9 ± 6.5%), medium (65.5 ± 6.6%), and high (93.7 ± 6.0%).

### 2.2. Statistical Analyses

Firstly, normality assumptions were checked for each group of players in each condition (i.e., national vs. foreign) using the Kolmogorov-Smirnov test. Secondly, a linear regression model was used to calculate the player’s contribution to the team’s performance for each game-related statistic as follows:

Variable x = β0 + β1 * Player’s performance variable x + εi,(1)

where β0 is the intercept, β1 is the impact of each predictor independent variable, and εi is the disturbance term. 

Subsequently, the predictor model was used to calculate the individual players’ performance for each variable with the constant (β0) divided by 5 to account for the number of players on court at one time (i.e., 5) in each model [19].

Thirdly, player’s contribution to the team was then examined via discriminant analysis to identify the game-related statistics that best discriminated between national and foreign players according to team ability. The structural coefficient (SC) above |0.30| identified the variables that best contributed to the differentiation of national and foreign players. Validation of each analysis was conducted using the leave-one-out classification models process [20]. All statistical analyses were conducted using PASW statistics 20 software (SPSS Inc., Chicago, IL, USA). The level of statistical significance was set at *p* < 0.05.

## 3. Results

The means and standard deviations with univariate differences between national and foreign players for each game-related statistic per playing position and team ability are presented in Table 1, Table 2 and Table 3. The univariate analysis showed that foreign players exhibited greater values for most game-related statistics compared to national players based upon Guard, Forward, and Center playing positions (see Table 1, Table 2 and Table 3).

The discriminant functions that differentiated national and foreign players were all statistically significant for each team ability group (*p* ≤ 0.05), with the structural coefficients for each playing position and team ability displayed in Table 4. The classification results for all functions were good to very good, with values greater than 69% for correct classification of cases [20].

When considering Guards, foreign players were discriminated from national players by % successful free-throws, % defensive rebounds, and % assists for all team ability clusters (i.e., poor, low, medium, and high, see Table 4). Specifically, foreign Guards exhibited higher values for % successful free-throws and % assists, while lower values for % defensive rebounds compared to national Guards (see Table 1). In addition, the variables % unsuccessful 2-point field-goals (lower values for foreign players, see Table 1) and minutes played (higher values for foreign players, see Table 1) discriminated foreign and national Guards for all team ability clusters, except for the high level cluster (see Table 4).

When considering Forwards, foreign players were discriminated from national players by % minutes played and % successful and unsuccessful 2-point field-goals for all team ability clusters (see Table 4). Foreign players exhibited higher values for minutes played and % successful 2-point field-goals and lower values for % unsuccessful 2-point field-goals compared to national players (see Table 2). Additionally, foreign Forwards exhibited statistically higher values for % assists and % turnovers compared to national forwards (see Table 2) when discriminated in low, medium, and high team ability clusters (see Table 4).

For the Center playing position, foreign players were discriminated from national Centers by % minutes played and % successful 2-point field-goals for all investigated team ability clusters (see Table 4), with greater values for foreign players (see Table 3). Furthermore, foreign Centers exhibited lower values of % unsuccessful 2-point field-goals and % defensive rebounds compared to national Centers (see Table 3) when discriminated in poor, low, and medium team ability clusters (see Table 4). Conversely, in the same team ability clusters (see Table 4), foreign players exhibited higher values of % offensive rebounds (see Table 4).

## 4. Discussion

The purpose of the present study was to identify the game-related statistics best discriminating national and foreign female basketball players according to playing positions and team ability. From a general point of view, the results showed that foreign players have a better performance according to team ability and playing position for most of the performance indicators, with higher values specifically for % minutes played, % successful 2-point field-goal, % successful free-throws, and % assists. These findings will be of extreme importance for coaches and managers when recruiting players based on their performance strengths and weaknesses, their playing position, and nationality [14,15].

This investigation provided a better understanding about the game performance of foreign and national female basketball players. Specifically, foreign Guards had better performance in % successful free-throws and % assists compared to national players. These results reflect the importance of foreign players during collaborative actions and drawing fouls since assists and free-throws, respectively, are considered two of the main KPIs in female basketball success [21,22]. One interesting finding from our results was the questionable differences for 2- and 3-point field-goals for Guards, where there was no consistent evidence for all clustered groups, despite this offensive match metric being a prominent focus for the playing position [11]. This finding indicated that Guards with similar, and likely very good, field-goal shooting ability were recruited within the Euroleague, irrespective of nationality. Conversely, foreign Guards had lower % defensive rebounds compared to national players, which is indicative of a greater offensive focus for foreign players compared to national players who were more defensive orientated. Subsequently, these greater skills (i.e., shooting, ball-handling, team-work, and assisting) should be primarily considered when recruiting foreign players to complement national players in the assembly of a successful team.

While the previously discussed variables differentiated national and foreign Guards regardless of team ability, the variable % minutes played discriminated between foreign and national players within the poor to medium clusters only. These findings indicated that lower level teams were more dependent on foreign Guards playing for success compared to national Guards. A possible explanation for this result might be due to the perceived regard of foreign players’ ability by coaches and teams [15] and typically greater financial investment in such players [14]. Further, it may be a result of the poorer shooting percentage (% unsuccessful 2-point field-goals) in national players and greater 3-point shooting by foreign players in these clusters (See Table 2), which are key statistics for success in female basketball [3,23]. Conversely, no differences were found in % minutes played between national and foreign Guards for the high cluster group. Possibly, high-level teams rely less on foreign Guards as a dominant role as teams recruit quality national Guards, which enables greater flexibility in this position for the team. Overall, these results highlight the importance of foreign Guards across all clusters, with greater reliance on these players to provide unique contributions to team success within lower team ability levels.

For the Forwards, a better 2-point shooting performance for foreign players compared to national players was identified for each team ability cluster, which matched with the higher % unsuccessful 2-point field-goals for national compared to foreign players. These results again confirm the importance of foreign players to team success as 2-point shooting ability has been highlighted as a fundamental performance indicator that differentiates winning and losing female basketball teams [4,24]. Foreign players in this position also accumulated more playing time compared to national Forwards across all team ability clusters, further highlighting teams’ reliance on these foreign players. This reliance was also reflected in the greater values of % assist and % turnovers for foreign players within the low, medium, and high team ability clusters. Due to the higher number of minutes played by foreign players compared to national players, foreign Forwards were likely able to perform more effective actions prior to scoring (i.e., assists), which is another key performance indicator in basketball [5,25,26,27,28]. Additionally, foreign Forwards performed a higher % of turnovers compared to national players, most likely due to their involvement in a greater number of ball possessions at the low-high team ability levels. Despite low turnovers being an important indicator that is able to discriminate between winning and losing elite female teams [5,24], the current results may indicate a potential risky behaviour of foreign players that may impact team performance and success. Coaches should consider this possible risky behaviour when considering foreign Forwards during recruitment.

Similar to the other playing position results, higher values for % minutes played, % successful 2-point field-goals, and % offensive rebounds were observed for foreign Centers (see Table 4). These results indicated a greater offensive ability for foreign Centres compared to national Centers. Further, national Centers exhibited poorer offensive (greater % unsuccessful 2-point field goals) and greater defensive (% defensive rebounds) actions, with these differences most notable at the lower team ability levels (poor-medium). National Centers also exhibited a greater defensive action (% blocks) compared to foreign Centers within the high team ability cluster. These results indicate that foreign and national Centers had distinct and different primary roles within the Euroleague, despite all aligning with the main offensive and defensive actions reported for Centers [11,21]. This distinction further highlighted the unique foci and use of foreign players within the Euroleague Women’s competition, with coaches and teams encouraged to consider this focus when developing team rosters. In fact, these results may reflect: (i) an improvement of more assertive and defensive behaviours for national players (higher commitment to their team) due to the recruitment of highly skilled foreign players for their offensive skills which national players compete/train against [29]; and (ii) the supplementary recruitment process of coaches and managers for national players to complement the skills and abilities of foreign players in this specific playing position [15].

To the best of our knowledge, this is the first study analysing the differences in game-related statistics between national and foreign elite female players according to different playing positions and team abilities. This novelty makes comparisons with other studies difficult, with some limitations needing discussion for future directions. Firstly, only games from the initial phase of the Euroleague Women’s competition were analysed for this study. Future studies might provide additional insight about the differences in game-related statistics between national and foreign female basketball players during the final competition phase, where game metrics were reported to be important for match success and different to initial competition phases [4,5]. Secondly, only the top European female basketball league was investigated, providing results for a specific basketball population, therefore future investigations are encouraged to examine other top leagues worldwide such as the WNBA. Finally, this study only examined one year of game statistics and it would be interesting to see if these differences between national and foreign players are stable or evolve over time. Future studies of larger datasets across multiple years will confirm the enduring impact of foreign and national female players on team performance to assist coaches with effective recruitment processes.

## 5. Conclusions

The discriminatory power of game-related statistics allowed us to identify the unique performances that national and foreign players exhibit according to team ability and playing position. Specifically, the main results revealed that foreign players exhibited higher values for: (i) playing time; (ii) shooting performances for free-throws and 2-point field-goals for the majority of clustered playing positions; (iii) team work (i.e., assists) for Guard and Forward players; (iv) securing defensive (i.e., Guards) and offensive rebounds (Centers); and (v) turnovers for Forwards. These results provide more information for managers to consider when recruiting national and foreign athletes for successful, elite female basketball teams. Further, these results will help coaches to better prepare technical-tactical training sessions so that individualized work can resolve specific and important vulnerabilities for greater player performance during competition. 

## Figures and Tables

**Table 1:** Descriptive results (M, mean; SD, standard deviation) of national and foreign Guards based upon poor, low, medium, and high team ability.

**Variables**	**Team Ability**									
	**Poor**					**Low**				
	**National**		**Foreign**		**Sig.**	**National**		**Foreign**		**Sig.**
	**M**	**SD**	**M**	**SD**		**M**	**SD**	**M**	**SD**	
Minutes	63082	35935	108453	26853	<0.01	75479	38092	95881	38527	<0.01
% Successful 2 pt	9.24	2.37	12.94	3.38	<0.01	10.42	2.94	11.37	3.53	<0.01
% Unsuccessful 2 pt	17.10	1.63	14.24	2.39	<0.01	16.57	1.69	15.79	2.29	<0.01
% Successful 3 pt	12.10	2.19	12.86	2.95	0.04	12.62	2.71	14.18	3.74	<0.01
% Unsuccessful 3 pt	14.03	0.26	14.02	0.30	0.70	14.08	0.24	13.87	0.33	<0.01
% Successful free throws	12.12	0.75	13.78	2.03	<0.01	12.53	1.14	13.43	1.88	<0.01
% Unsuccessful free throws	13.87	0.04	13.89	0.07	<0.01	13.88	0.05	13.89	0.05	0.17
% Offensive rebounds	13.24	0.29	13.40	0.38	<0.01	13.29	0.31	13.34	0.35	0.15
% Assists	8.67	1.62	9.74	1.84	<0.01	9.02	1.46	9.79	1.89	0.00
% Steals	13.07	0.80	13.76	1.26	<0.01	13.31	1.13	13.61	1.11	0.01
% Defensive rebounds	13.38	0.56	12.92	0.72	<0.01	13.30	0.63	12.96	0.72	<0.01
% Fouls	15.04	1.37	15.82	1.40	<0.01	15.06	1.30	15.16	1.28	0.44
% Turnover	14.49	1.03	15.04	1.11	<0.01	14.53	0.92	14.97	1.23	<0.01
% Blocks	13.71	0.41	13.54	0.61	0.02	13.69	0.51	13.61	0.58	0.13
**Variables**	**Medium**					**High**				
	**National**		**Foreign**		**Sig.**	**National**		**Foreign**		**Sig.**
	**M**	**SD**	**M**	**SD**		**M**	**SD**	**M**	**SD**	
Minutes	67214	29659	108157	22683	<0.01	67406	36273	73706	22327	0.22
% Successful 2 pt	9.68	2.39	11.79	4.41	<0.01	11.08	3.42	10.63	2.55	0.39
% Unsuccessful 2 pt	16.81	1.41	15.71	1.75	<0.01	16.68	1.59	16.84	1.16	0.51
% Successful 3 pt	12.39	2.27	13.60	3.05	<0.01	12.75	2.86	14.35	4.73	0.04
% Unsuccessful 3 pt	14.11	0.19	13.94	0.24	<0.01	14.06	0.24	14.05	0.32	0.93
% Successful free throws	12.54	1.20	13.50	1.95	<0.01	12.53	1.16	13.17	1.74	0.03
% Unsuccessful free throws	13.87	0.04	13.90	0.06	<0.01	13.87	0.03	13.88	0.04	0.47
% Offensive rebounds	13.30	0.29	13.37	0.35	0.11	13.20	0.17	13.27	0.29	0.13
% Assists	9.02	1.72	10.48	2.13	<0.01	8.84	1.23	9.41	1.38	0.02
% Steals	13.46	1.06	13.72	0.96	0.09	13.26	0.88	13.36	0.96	0.58
% Defensive rebounds	13.34	0.57	12.90	0.71	<0.01	13.55	0.43	13.14	0.76	<0.01
% Fouls	15.04	1.24	15.63	1.26	<0.01	14.46	1.14	14.81	1.11	0.08
% Turnover	14.45	0.78	15.14	1.11	<0.01	14.14	0.62	14.38	0.82	0.09
% Blocks	13.76	0.31	13.64	0.51	0.04	13.65	0.60	13.68	0.52	0.81

**Table 2:** Descriptive results (M, mean; SD, standard deviation) of national and foreign Forwards based upon poor, low, medium, and high team ability.

**Variables**	**Team Ability**									
	**Poor**					**Low**				
	**National**		**Foreign**		**Sig.**	**National**		**Foreign**		**Sig.**
	**M**	**SD**	**M**	**SD**		**M**	**SD**	**M**	**SD**	
Minutes	54963	23559	105630	13713	<0.01	70839	40331	93701	36665	<0.01
% Successful 2 pt	8.91	1.61	12.77	3.45	<0.01	10.38	3.64	13.15	4.37	<0.01
% Unsuccessful 2 pt	17.05	1.40	14.34	2.06	<0.01	16.79	1.85	15.62	1.96	<0.01
% Successful 3 pt	11.37	1.78	12.70	2.63	0.01	12.84	2.88	12.80	3.10	0.93
% Unsuccessful 3 pt	14.17	0.17	14.08	0.27	0.08	14.00	0.37	14.02	0.34	0.80
% Successful free throws	12.31	1.06	13.11	1.76	0.02	12.73	1.78	13.19	1.54	0.04
% Unsuccessful free throws	13.87	0.02	13.88	0.04	0.13	13.89	0.06	13.88	0.05	0.59
% Offensive rebounds	13.25	0.27	13.69	0.66	<0.01	13.41	0.43	13.66	0.54	<0.01
% Assists	7.99	0.79	8.44	1.21	0.07	8.20	1.14	8.72	1.17	<0.01
% Steals	12.76	0.41	13.45	0.67	<0.01	13.37	1.09	13.57	1.25	0.21
% Defensive rebounds	13.43	0.43	12.43	0.90	<0.01	13.08	0.92	12.94	0.82	0.24
% Fouls	15.43	1.31	16.09	1.24	0.04	14.86	1.24	15.45	1.33	<0.01
% Turnover	14.21	0.68	14.77	0.94	0.01	14.28	0.82	14.73	1.02	<0.01
% Blocks	13.67	0.78	13.31	0.76	0.06	13.56	0.63	13.37	0.87	0.07
**Variables**	**Medium**					**High**				
	**National**		**Foreign**		**Sig.**	**National**		**Foreign**		**Sig.**
	**M**	**SD**	**M**	**SD**		**M**	**SD**	**M**	**SD**	
Minutes	33258	18751	96918	33552	<0.01	31552	23689	111269	19188	<0.01
% Successful 2 pt	8.37	1.53	13.07	4.06	<0.01	8.51	1.75	15.33	4.93	<0.01
% Unsuccessful 2 pt	17.57	1.04	15.28	2.20	<0.01	17.98	0.88	14.20	1.99	<0.01
% Successful 3 pt	12.21	2.30	13.26	3.55	<0.01	11.89	2.14	13.38	2.79	<0.01
% Unsuccessful 3 pt	14.18	0.19	14.06	0.29	<0.01	14.19	0.16	14.07	0.20	<0.01
% Successful free throws	12.37	1.07	13.58	2.01	<0.01	12.27	0.84	14.13	1.99	<0.01
% Unsuccessful free throws	13.87	0.03	13.90	0.06	<0.01	13.87	0.03	13.91	0.05	<0.01
% Offensive rebounds	13.27	0.30	13.78	0.65	<0.01	13.30	0.33	13.69	0.72	<0.01
% Assists	7.74	0.62	8.49	1.05	<0.01	7.78	0.70	10.15	1.63	<0.01
% Steals	12.78	0.44	13.47	0.96	<0.01	12.79	0.57	13.93	1.27	<0.01
% Defensive rebounds	13.56	0.43	12.38	1.10	<0.01	13.48	0.48	11.87	1.11	<0.01
% Fouls	14.47	1.18	15.26	1.22	<0.01	14.14	1.07	15.38	1.08	<0.01
% Turnover	13.87	0.53	14.69	1.02	<0.01	13.81	0.37	14.86	0.96	<0.01
% Blocks	13.70	0.43	12.74	1.75	<0.01	13.71	0.42	13.13	1.32	<0.01

**Table 3:** Descriptive results (M, mean; SD, standard deviation) of national and foreign Centers based upon poor, low, medium, and high team ability.

**Variables**	**Team Ability**									
	**Poor**					**Low**				
	**National**		**Foreign**		**Sig.**	**National**		**Foreign**		**Sig.**
	**M**	**SD**	**M**	**SD**		**M**	**SD**	**M**	**SD**	
Minutes	23093	20769	99350	25994	<0.01	71115	34005	92214	29669	<0.01
% Successful 2 pt	8.06	1.18	14.78	4.22	<0.01	11.37	3.73	14.82	4.71	<0.01
% Unsuccessful 2 pt	17.78	1.10	14.78	2.06	<0.01	15.84	1.93	14.60	2.12	<0.01
% Successful 3 pt	10.82	0.49	11.74	2.33	0.02	11.66	2.11	11.65	2.26	0.94
% Unsuccessful 3 pt	14.30	0.04	14.14	0.28	<0.01	14.19	0.17	14.20	0.20	0.64
% Successful free throws	11.98	0.55	14.03	2.19	<0.01	12.77	1.41	13.27	1.83	<0.01
% Unsuccessful free throws	13.87	0.04	13.89	0.09	0.22	13.89	0.06	13.90	0.07	0.03
% Offensive rebounds	13.21	0.22	13.81	0.64	<0.01	13.51	0.47	13.80	0.77	<0.01
% Assists	7.47	0.31	8.31	0.95	<0.01	8.41	1.10	8.28	0.91	0.23
% Steals	12.77	0.47	13.15	0.79	0.01	13.32	0.99	13.28	0.97	0.69
% Defensive rebounds	13.70	0.43	12.17	1.17	<0.01	12.83	0.86	12.32	1.11	<0.01
% Fouls	14.65	1.33	15.43	1.15	<0.01	15.46	1.41	15.65	1.30	0.19
% Turnover	13.88	0.56	14.71	0.87	<0.01	14.52	0.89	14.62	0.92	0.28
% Blocks	13.73	0.37	12.79	1.57	<0.01	13.16	1.24	13.23	1.03	0.58
**Variables**	**Medium**					**High**				
	**National**		**Foreign**		**Sig.**	**National**		**Foreign**		**Sig.**
	**M**	**SD**	**M**	**SD**		**M**	**SD**	**M**	**SD**	
Minutes	51562	28634	97377	20977	<0.01	55396	24043	79901	21390	<0.01
% Successful 2 pt	11.05	3.87	16.23	4.47	<0.01	12.10	4.30	16.08	4.80	<0.01
% Unsuccessful 2 pt	16.64	1.39	14.52	2.18	<0.01	16.02	1.98	15.45	1.89	0.16
% Successful 3 pt	11.74	2.45	11.47	1.96	0.47	10.73	0.00	11.16	1.34	0.04
% Unsuccessful 3 pt	14.19	0.22	14.18	0.22	0.87	14.30	0.03	14.27	0.08	0.01
% Successful free throws	12.54	1.04	13.30	1.68	<0.01	12.69	1.16	13.58	1.92	0.01
% Unsuccessful free throws	13.88	0.06	13.92	0.08	0.01	13.90	0.07	13.89	0.05	0.54
% Offensive rebounds	13.37	0.32	13.96	0.74	<0.01	13.85	0.67	13.83	0.56	0.86
% Assists	7.73	0.60	8.66	1.10	<0.01	8.08	0.88	8.32	0.84	0.18
% Steals	12.84	0.47	13.35	0.91	<0.01	13.06	0.65	13.44	1.01	0.04
% Defensive rebounds	13.28	0.72	12.14	0.99	<0.01	12.84	0.90	12.64	0.79	0.25
% Fouls	14.87	1.35	15.11	1.31	0.28	15.29	1.47	15.30	1.39	0.98
% Turnover	14.05	0.63	14.66	0.82	<0.01	14.03	0.55	14.17	0.76	0.31
% Blocks	13.22	1.24	12.81	1.43	0.08	12.66	1.60	11.58	2.19	0.01

**Table 4:** Discriminant function structure coefficients (SC) and tests of significance for each game metric that differentiated foreign and national Guards, Forwards, and Centers, based upon poor, low, medium, and high team ability.

Variables	Guards				Forwards				Centers			
	Poor	Low	Med	High	Poor	Low	Med	High	Poor	Low	Med	High
	SC	SC	SC	SC	SC	SC	SC	SC	SC	SC	SC	SC
Minutes	0.68	−0.52	0.84	0.21	0.70	0.55	0.91	0.72	0.90	−0.59	0.80	0.66
% Successful 2 pt	0.64	−0.29	0.34	−0.14	0.43	0.64	0.59	0.40	0.54	−0.74	0.51	0.53
% Unsuccessful 2 pt	−0.71	0.39	−0.39	0.11	−0.45	−0.57	−0.52	−0.53	−0.48	0.56	−0.47	−0.18
% Successful 3 pt	0.15	−0.49	0.25	0.35	0.17	−0.01	0.14	0.12	0.14	0.01	−0.05	0.27
% Unsuccessful 3 pt	−0.03	0.75	−0.45	−0.02	−0.12	0.03	−0.20	−0.13	−0.19	−0.04	−0.01	−0.36
% Successful free throws	0.57	−0.60	0.34	0.37	0.16	0.26	0.29	0.26	0.32	−0.28	0.22	0.34
% Unsuccessful free throws	0.21	−0.13	0.28	0.12	0.10	−0.07	0.22	0.18	0.07	−0.20	0.19	−0.08
% Offensive rebounds	0.24	−0.14	0.13	0.25	0.26	0.47	0.39	0.15	0.32	−0.42	0.40	−0.02
% Assists	0.30	−0.46	0.42	0.39	0.13	0.42	0.34	0.41	0.30	0.11	0.41	0.17
% Steals	0.33	−0.27	0.14	0.09	0.36	0.16	0.36	0.25	0.16	0.04	0.28	0.27
% Defensive rebounds	−0.36	0.49	−0.38	−0.56	−0.42	−0.15	−0.55	−0.40	−0.44	0.46	−0.54	−0.15
% Fouls	0.28	−0.08	0.26	0.29	0.14	0.43	0.26	0.23	0.19	−0.12	0.08	0.00
% Turnover	0.26	−0.41	0.40	0.29	0.20	0.45	0.39	0.31	0.30	−0.10	0.34	0.13
% Blocks	−0.17	0.15	−0.17	0.04	−0.13	−0.23	−0.29	−0.13	−0.21	−0.05	−0.13	−0.35
Eigenvalue	1.020	0.248	0.823	0.278	3.211	0.296	1.658	6.177	2.625	0.307	1.342	0.678
% of Variance	100	100	100	100	100	100	100	100	100	100	100	100
Canonical Correlation	0.711	0.446	0.672	0.467	0.873	0.478	0.79	0.928	0.851	0.484	0.757	0.636
Wilks’ Lambda	0.495	0.801	0.548	0.782	0.237	0.772	0.376	0.139	0.276	0.765	0.427	0.596
Chi-square	129.409	92.869	108.705	30.215	86.262	54.932	122.18	206.948	133.941	101.884	116.588	43.455
df	14	14	14	14	14	14	14	14	14	14	14	14
Sig.	<0.001	<0.001	<0.001	0.007	<0.001	<0.001	<0.001	<0.001	<0.001	<0.001	<0.001	<0.001
Classification results %	83.9	70.0	81.1	69.7	100	73.8	91.0	99.1	92.0	70.8	84.9	80.6
